# Vitamin D and Aging: Central Role of Immunocompetence

**DOI:** 10.3390/nu16030398

**Published:** 2024-01-30

**Authors:** Carsten Carlberg, Eunike Velleuer

**Affiliations:** 1 Institute of Animal Reproduction and Food Research, Polish Academy of Sciences, PL-10-748 Olsztyn, Poland; 2 School of Medicine, Institute of Biomedicine, University of Eastern Finland, FI-70211 Kuopio, Finland; 3 Department for Cytopathology, Heinrich-Heine-University Düsseldorf, D-40225 Düsseldorf, Germany; velleuer@uni-duesseldorf.de; 4 Department for Pediatric Hemato-Oncology, Helios Children’s Hospital, D-47805 Krefeld, Germany

**Keywords:** vitamin D, aging, innate immunity, immunocompetence, hematopoiesis, epigenome, transcriptome, vitamin D target genes

## Abstract

The pro-hormone vitamin D_3_ is an important modulator of both innate and adaptive immunity since its biologically active metabolite 1α,25-dihydroxyvitamin D_3_ (1,25(OH)_2_D_3_) regulates via the transcription factor VDR (vitamin D receptor) the epigenome and transcriptome of human immune cells and controls in this way the expression of hundreds of vitamin D target genes. Since the myeloid linage of hematopoiesis is epigenetically programmed by VDR in concert with the pioneer factors PU.1 (purine-rich box 1) and CEBPα (CCAAT/enhancer binding protein α), monocytes, macrophages, and dendritic cells are the most vitamin D-sensitive immune cell types. The central role of the immune system in various aging-related diseases suggests that immunocompetence describes not only the ability of an individual to resist pathogens and parasites but also to contest non-communicative diseases and the process of aging itself. In this review, we argue that the individual-specific responsiveness to vitamin D relates to a person’s immunocompetence via the epigenetic programming function of VDR and its ligand 1,25(OH)_2_D_3_ during hematopoiesis as well as in the periphery. This may provide a mechanism explaining how vitamin D protects against major common diseases and, in parallel, promotes healthy aging.

## 1. Introduction

Vitamin D has important endocrine functions that are often defined by its deficiency, which most prominently causes a bone phenotype that is manifested as rickets in children and osteomalacia in adults [1]. The physiological basis of this observation is that vitamin D has, via its role as a controller of calcium homeostasis, a key function in bone formation [2]. In contrast, in other physiological processes, such as the modulation of immunity, the role of vitamin D is not as unique as concerning the regulation of serum calcium levels, and therefore a lack of vitamin D can be compensated, at least in part, by other regulatory molecules [3]. This also partly explains why large trials like VITAL (VITamin D and OmegA-3 TriaL) and ViDA (Vitamin D Assessment) had a null result as the primary outcome concerning the non-skeletal effects of vitamin D_3_ supplementation [4,5].

**Figure 1:**
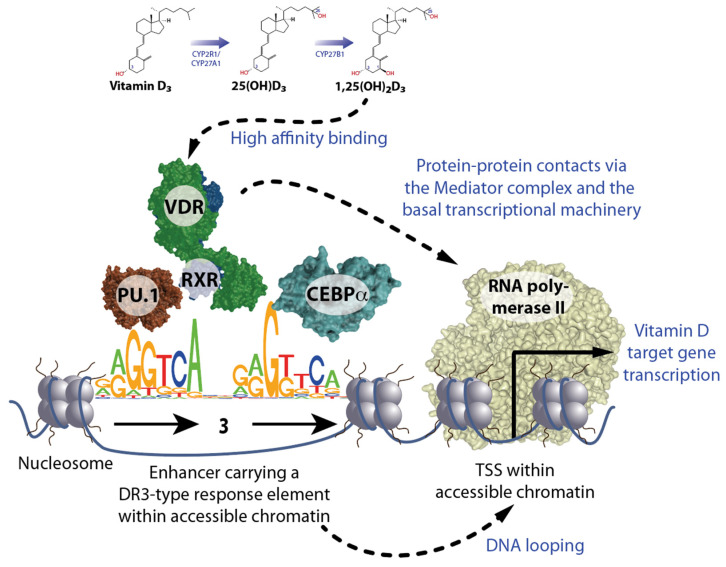
Principles of vitamin D signaling. The biologically most active vitamin D_3_ metabolite 1,25(OH)_2_D_3_ activates at sub-nanomolecular concentrations the transcription factor VDR, which, together with its coreceptor RXR, preferentially contacts DR3-type sequences. The opening of chromatin at DR3-type binding sites carrying enhancer regions is supported by the pioneer factors PU.1 and CEBPα. The looping of enhancers to TSS regions facilitates protein–protein contacts in activated VDR via the Mediator complex and the basal transcriptional machinery with RNA polymerase II. This modulates target gene transcription.

Vitamin D deficiency is primarily related to a modern lifestyle that is characterized by insufficient exposure of bare skin to the UV-B component of sunlight. The latter is essential for the endogenous production of vitamin D_3_ from 7-dehydrocholesterol in the skin [6,7]. Vitamin D_3_ itself has no biological function in the human body, but when it is converted in the liver through the enzymes CYP (cytochrome P450) 2R1 and CYP27A1 to 25-hydroxyvitamin D_3_ (25(OH)D_3_) and further in the kidneys by the enzyme CYP27B1 to 1,25(OH)_2_D_3_, it acts as a hormone [8] (Figure 1, top). Interestingly, cells of the innate immune system, such as monocytes, macrophages, and dendritic cells, as well as skin and bone cells, express the *CYP27B1* gene and are able to produce 1,25(OH)_2_D_3_ for auto- and paracrine purposes [9]. In an evolutionary adaptation process, 1,25(OH)_2_D_3_ became, some 550 million years ago, a high-affinity ligand for the nuclear receptor VDR [10]. Like other members of the nuclear receptor superfamily, such as estrogen receptors (ESRs) and glucocorticoid receptors (GR), VDR functions as a transcription factor and controls the expression of hundreds of vitamin D target genes (more details in Section 2). Thus, 1,25(OH)_2_D_3_ is an endocrine compound that has an impact on human physiology comparable to that of estrogen and cortisol. This concept of a pleiotropic action of vitamin D is supported by the observation that the *VDR* gene is significantly expressed in most human tissues and cell types, i.e., the function of VDR seems not to be restricted to the intestine for controlling the resorption of calcium.

The first hints of the immunomodulatory effect of vitamin D arose at about the same time, when vitamin D_3_ supplementation was found to prevent experimentally created rickets [11,12]. Cod liver oil, which is high in vitamin D_3_, as well as UV-B exposure, were both used for the protection and treatment of tuberculosis, i.e., against an infectious disease caused by intracellular bacteria [13,14]. Furthermore, the risk of autoimmune diseases, such as multiple sclerosis, was found to be reduced by a sufficient vitamin D status [15]. Thus, vitamin D has obvious immune regulatory functions, which in fact are evolutionary older than the control of calcium homeostasis [2].

The vitamin D status is defined by the serum 25(OH)D_3_ level, which should be in a range of 75–150 nM (30–60 ng/mL) [16]. Individuals can be segregated by their vitamin D status into deficient (below a serum 25(OH)D_3_ level of 30 nM), insufficient (30–74.9 nM), and sufficient (75–150 nM). This parameter reflects the endogenous production of vitamin D_3_ and/or its supplementation via diet or pills and majorly depends on the lifestyle decisions of the individual. In addition, people were found to be high, mid, or low responders to vitamin D, i.e., they differ in the efficiency of their molecular response to vitamin D [17]. This vitamin D response index is based on variations of both the genome and the epigenome, i.e., in contrast to the vitamin D status, the index does not change depending on season, diet, and/or supplementation [18]. It is assumed that low vitamin D responders, who represent about 25% of the population, have a higher susceptibility to diseases, in particular those that are related to a compromised immune system, like multiple sclerosis [19].

In this review, we will discuss how VDR and its ligand 1,25(OH)_2_D_3_ modulate the immunocompetence of individuals and how the latter declines during aging. This concept can not only explain how vitamin D affects the rate of aging but also how it reduces the risk of many age-related diseases.

## 2. Principles of Vitamin D Signaling

The more than 1600 transcription factors encoded by the human genome bind sequence-specifically to genomic DNA [20]. The specific DNA binding motif of VDR is the sequence RGKTSA (R = A or G, K = G or T, and S = C or G). In a heterodimeric complex with the nuclear receptor RXR (retinoid X receptor), VDR binds preferentially to a direct repeat of this sequence motif with a spacing of 3 base pairs (bp) (DR3) [21,22,23] (Figure 1, bottom left). A pre-condition for efficient recognition of such DR3-type response elements by VDR-RXR heterodimers is their location within accessible open chromatin (euchromatin). Since more than 90% of genomic DNA is located within closed heterochromatin, VDR often uses the help of pioneer factors to open chromatin. Pioneer factors are transcription factors that have very short DNA recognition sequences, which they bind even in the presence of a nucleosome [24]. The latter are every 200 bp repeating subunits of chromatin that are composed of pairs of the histone proteins H2A, H2B, H3, and H4, around which 147 bp of genomic DNA are wrapped nearly twice. While in immune cells VDR is supported by the transcription factors PU.1 and CEBPα [25], in osteoblasts these are RUNX2 (RUNX family transcription factor 2) and CEBPα [26], and in T cells BACH2 (BTB domain and CNC homolog 2) [27].

Next-generation sequencing technologies, such as FAIRE-seq (formaldehyde-assisted isolation of regulatory elements sequencing that is nowadays mostly replaced by ATAC-seq (assay for transposase-accessible chromatin using sequencing)) and ChIP-seq (chromatin immunoprecipitation sequencing), are able to monitor in a genome-wide fashion the accessibility of chromatin or the binding of VDR to genomic DNA, respectively [28]. PBMCs (peripheral blood mononuclear cells) are a natural mixture of T cells, B cells, NK (natural killer) cells, ILCs (innate lymphoid cells), and monocytes [29]. They are primary cell types that can be isolated with minimal harm to the donor, and their use in human vitamin D_3_ supplementation studies allows the assessment of vitamin D signaling under in vivo conditions.

**Figure 2:**
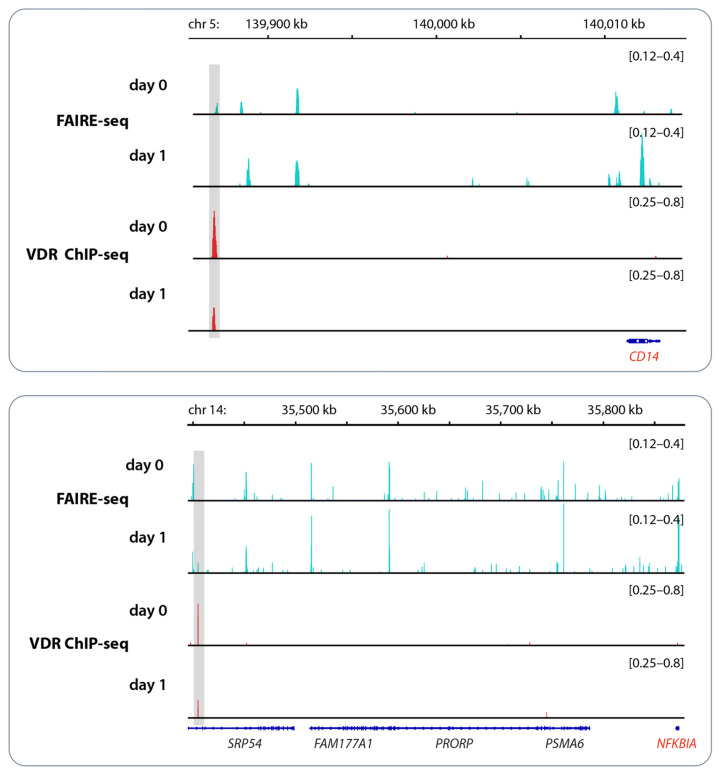
Example of vitamin D signaling in vivo. Chromatin opening and VDR binding at the loci of the genes *CD14* (**top**) and *NFKBIA* (**bottom**) measured by FAIRE-seq and ChIP-seq, respectively, in PBMCs obtained from an individual who was challenged with a vitamin D_3_ bolus (2000 μg). PBMCs were isolated directly before (day 0) and 24 h after (day 1) vitamin D_3_ supplementation without any further in vitro culture. The peak tracks represent the merger of each of the three biological repeats [31]. Enhancer regions with VDR binding sites are shaded in gray, and vitamin D target genes are highlighted in red. Please note that the FAIRE-seq data indicate far more vitamin D-triggered accessible chromatin regions than VDR-binding enhancers.

In the example of the genomic regions of the vitamin D target genes *CD14* (CD14 molecule) and *NFKBIA* (NFκB inhibitor alpha), changes in chromatin accessibility and VDR binding in response to vitamin D_3_ bolus supplementation are demonstrated (Figure 2). The VDR binding enhancer of the *CD14* gene is located 26 kb (kilo bp) downstream of the gene’s TSS (transcription start site), while for the *NFKBIA* gene, the enhancer region lies even 470 kb downstream. As long as the enhancer and the TSS region are located within the same TAD (topologically associating domain), they can efficiently interact by DNA looping. TADs are subunits of chromatin with an average size of approximately 1000 kb that are functionally insulated from each other by loop formation directed by the chromatin organizer CTCF (CCCTC-binding factor) [30]. This implies that 1000 kb is the maximal distance between a VDR-binding enhancer and the TSS of a gene affected by this regulatory element. Interestingly, in the in vivo experiment shown in Figure 2, VDR binding to both enhancer regions decreased after vitamin D_3_ supplementation [31]. This fits with the downregulation of the expression of both genes in response to vitamin D [32].

This example demonstrates that VDR-binding enhancers and TSS regions of target genes can be at a greater linear distance from each other since DNA looping brings them into close vicinity so that VDR can influence, via protein–protein interactions with the Mediator complex and the basal transcriptional machinery, the activity of RNA polymerase II (Figure 1, bottom right). These genomic actions of vitamin D take hours since new mRNA needs to be synthesized and translated into protein. In contrast, the rapid non-genomic actions of vitamin D have also been described [33], which may be mediated by membrane-associated proteins such as PDIA3 (protein disulfide isomerase family A member 3) [34,35]. However, epigenetic effects of vitamin D require the involvement of VDR, i.e., for the processes discussed here, such as hematopoiesis and immunocompetence, non-genomic effects are most likely not relevant.

Genome-wide patterns of DNA methylation, post-translational modification of histone proteins, and three-dimensional chromatin organization determine the epigenome, i.e., the so-called epigenetic landscape, of a cell [36]. In contrast to the genome, which should not change significantly during a lifetime, the epigenome dynamically responds to intra- and extracellular signals, including vitamin D and other nuclear hormones. Vitamin D does not only affect genome-wide the binding of some 20,000 VDR binding sites, the so-called VDR cistrome [28], and accessible chromatin [31], but also that of pioneer factors, CTCF, and histone markers of active chromatin [25,37]. For example, in the monocytic leukemia cell line THP-1, vitamin D affects the local epigenetic pattern in more than 500 TSS- and 2500 enhancer regions. Thus, vitamin D has in vitro as well as in vivo a modulatory role on human immune cells, such as THP-1 cells and PBMCs from healthy donors [18]. The epigenome-wide effects of vitamin D translate then, at least in part, into changes of the transcriptome as measured by RNA-seq (RNA sequencing) [38].

## 3. Vitamin D and Epigenetic Programming of Innate Immune Cells

**Figure 3:**
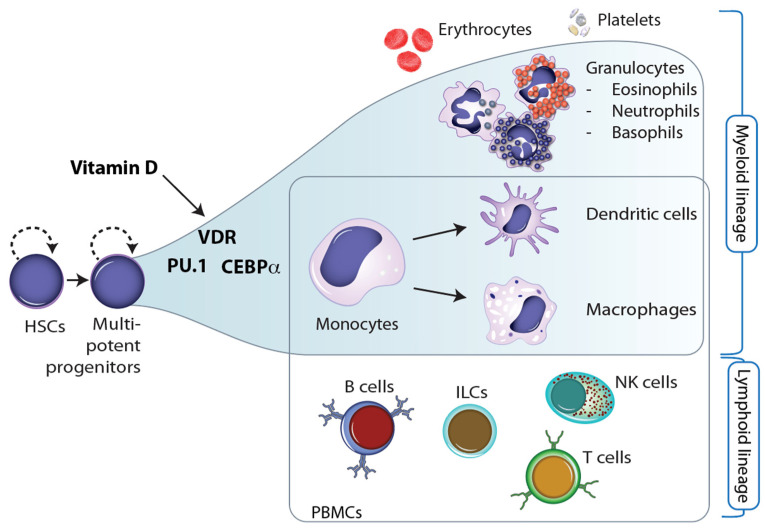
Vitamin D and hematopoiesis. Together with the pioneer factors PU.1 and CEBPα, VDR directs the differentiation of myeloid progenitor cells into monocytes and granulocytes. This may explain why monocytes and their derived cells, dendritic cells and macrophages, are the most vitamin D-responsive cell types of the immune system.

Throughout embryogenesis as well as during cellular differentiation in the tissues of adults, stem and progenitor cells undergo major changes in their epigenome that determine the function and properties of the outcome, i.e., of terminally differentiated cells [39]. The epigenetic landscape of these cells is reshaped so that lineage-specific genes are prominently expressed while genes of other cell lineages are repressed [40]. This epigenetic programming process is driven by specific sets of transcription factors and chromatin-modifying enzymes that determine, at bifurcation points, in which direction the cells are differentiating [41]. In the process of hematopoiesis, HSCs (hematopoietic stem cells), which are located in the bone marrow, differentiate into more than 100 cell types of the blood, such as erythrocytes and platelets, and the immune system, such as B and T cells, monocytes, NK cells, ILCs, neutrophils, and many more [42] (Figure 3).

Interestingly, 1,25(OH)_2_D_3_ has been shown to regulate the number of embryonic HSCs [43]. Moreover, VDR, PU.1, and CEBPα are the key transcription factors for the differentiation of myeloid progenitor cells into monocytes and granulocytes, i.e., into major cells of innate immunity [44]. This implies that vitamin D has a direct effect on the epigenetic programming of monocytes (Figure 3). This may explain why these innate immune cells are most responsive to vitamin D [45]. Moreover, vitamin D also affects the differentiation of monocytes into dendritic cells and macrophages. For example, the vitamin D target gene *TNFSF11* (TNF superfamily member 11, encoding for the cytokine RANKL) controls the differentiation of monocytes into bone-resorbing osteoclasts [46].

Epigenetic programming of differentiating cells is, in most cases, an irreversible process that, however, is modulated by many intrinsic and extrinsic factors [47]. This allows cells with a rapid turnover, in particular those of the innate immune system, to adapt to changes in environmental conditions, such as microbe infection, inflammation, and the onset of non-communicative diseases like cancer, diabetes, and neurodegeneration [48]. For example, it was shown that in vitamin D-sufficient mice, vitamin D-deficient fetal HSCs can induce diabetes [49]. In this mouse model, vitamin D deficiency epigenetically reprograms HSCs. Importantly, similar processes were found in vitamin D-deficient human monocytes. A further indication of the impact of VDR and 1,25(OH)_2_D_3_ on the differentiation of the myeloid line of hematopoiesis is the observation that high *VDR* gene expression is associated with a good prognosis of AML (acute myeloid leukemia) and that synthetic VDR ligands are promising disease-modifying drugs [50].

Monocytes not only control the inflammatory response of the body but also coordinate, via their derived cells, dendritic cells and macrophages, the response to many different types of stress. This specification of monocyte differentiation is part of the process of trained immunity [51,52], which is a form of epigenetic memory of immune challenges in the form of chromatin changes. This prepares the innate immune cells better for a possible next encounter with the same microbes. The cluster of *HLA* (human leukocyte antigen) genes also contains a larger number of other immunologically important genes [53], many of which are vitamin D targets [32]. Accordingly, the *HLA* gene cluster represents not only the most variant region of the human genome but is also a “hotspot” for the actions of vitamin D on the immune system.

Since individuals show differences both in their vitamin D status as well as in their vitamin D response index, they also differ in the epigenetic programming of monocytes and their derived cells during hematopoiesis [54]. Accordingly, optimized vitamin D_3_ supplementation may support both proper epigenetic programming of immune cells throughout hematopoiesis in the bone marrow as well as differentiation in tissues in response to antigen encounters [19]. Thus, we suggest that the main effect of vitamin D on the immune system is the epigenetic programming in central immune organs as well as in the periphery. This concept needs further experimental confirmation, but it provides an attractive model for explaining individuals’ differences in the responsiveness of their innate immune systems. Finally, this contributes to the immunocompetence of the individual.

## 4. Decline in Immunocompetence during Aging

Aging is a natural and unavoidable process of the accumulation of molecular and cellular damage, which leads to defective functions of cells, tissues, and whole organs that weaken the whole human body [55]. The process of aging is driven by 12 different hallmarks that relate either to (i) age-associated manifestation, (ii) the acceleration of aging, or (iii) deceleration or reversing aging [56]. Some of these hallmarks of aging, such as disabled macroautophagy, stem cell exhaustion, chronic inflammation, and dysbiosis, indicate profound alterations of the immune system during aging that contribute to a decline in immunocompetence [57].

**Figure 4:**
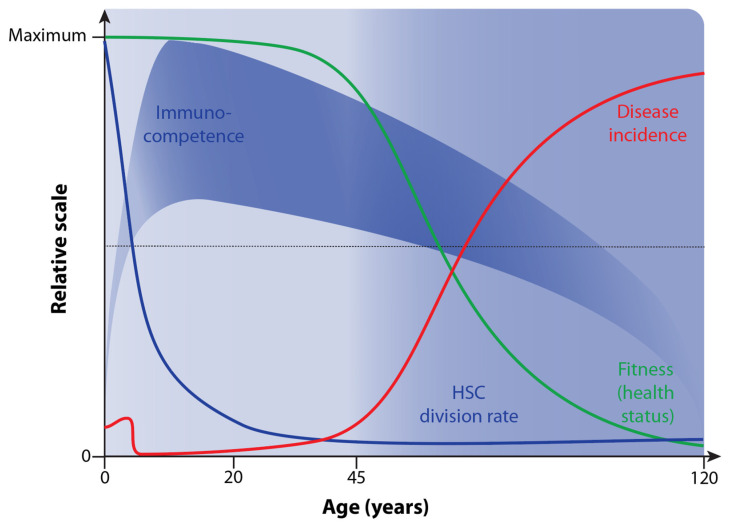
Changes in immunocompetence and HSC division rate over lifetime and their relation to health status/aging and disease incidence.

Immunocompetence is primarily defined as the ability of the human body to respond appropriately to antigen exposure [58]. In this way, invading microbes are efficiently cleared. In addition, in a “competent” immune system, there are no overreactions of immune cells that may lead to health- and life-threatening reactions, such as septic shock, anaphylactic responses, or autoimmune diseases. Accordingly, immunocompetence opposes immunodeficiency, which occurs in newborns but also as the result of diseases such as AIDS (acquired immunodeficiency syndrome) or immunosuppressive medication, e.g., after organ transplantation. The competence of the immune system drastically increases after birth and reaches its peak at the age of 10 [59] (Figure 4).

The thymus is a primary lymphoid organ in which immunocompetent T cells are produced. However, already at a young age, the cell mass, structure, and architecture of the thymus regress, and the number of naïve T cells produced declines [60]. In addition to thymus atrophy, the immune system deteriorates with age in many other ways, of which the decline of the HSC division rate [61] and increased rates of chronic inflammation, referred to as inflammaging, are most important [57,62]. This immunosenescence leads to increased susceptibility and higher incidences of a large variety of infectious diseases as well as non-communicative disorders like cancer, diabetes, and autoimmune diseases in older adults [63] (Figure 4).

**Figure 5:**
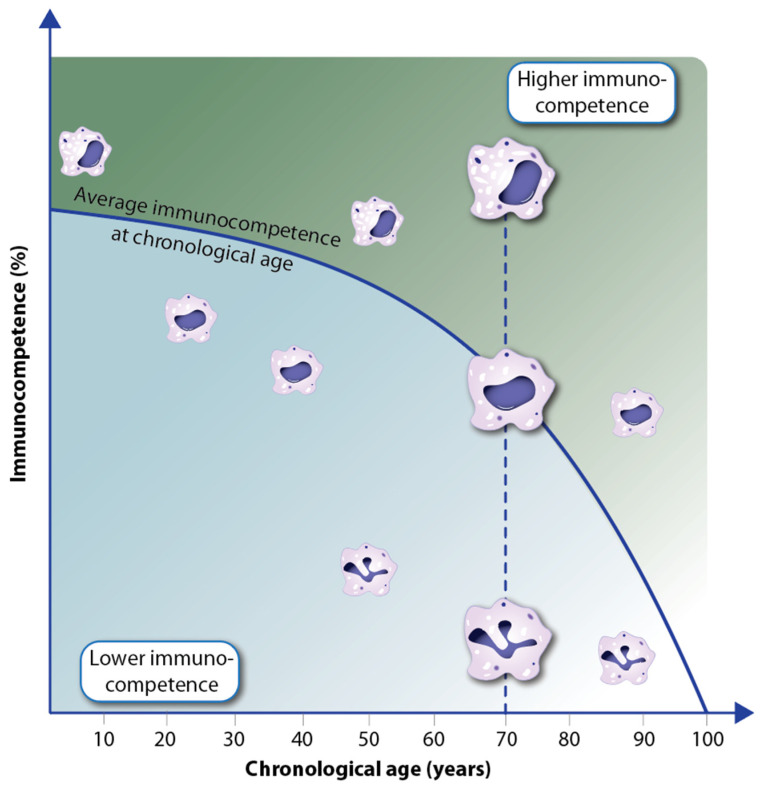
Individual decline of immunocompetence with aging. In the example of macrophages of different functionality (depicted via the integrity of the nucleus and the number of granules), interindividual differences in the immunocompetence of members of the same age cohort are expressed.

On the molecular and cellular level, immunocompetence is reflected in the different functionality of immune cells as well as their well-orchestrated interactions. For example, macrophages with a single intact nucleus and a large number of granules are more functional, e.g., in performing phagocytosis, than others with a polymorphic nucleus and a low number of granules (Figure 5). With declining overall immunocompetence during aging, the relative number of competent immune cells decreases [64]. However, there are interindividual differences, i.e., with increasing chronological age, there are people with a higher percentage of immunocompetent cells than the average and others with a lower number [65]. Thus, within the same age cohort, there are individuals with higher immune resilience and others with lower immunocompetence [66]. Accordingly, it can be assumed that in the first group, the rate of aging is slower and the incidence of diseases is lower, while in the latter group, accelerated aging and a higher disease rate should be observed. This concept fits with the observation of epigenetic clocks in various tissues of humans and other species [67]. Traditionally, these clocks are measured via the methylation levels of a few hundred genomic regions, which are chosen as representatives for epigenetic changes in the whole epigenome [68].

The decline in immunocompetence results in reduced immunosurveillance, such as the detection and destruction of neoplastic cells by cytotoxic T cells [69]. As a consequence, the cancer risk of persons with low immunocompetence is significantly higher than that of individuals with high immunocompetence [66]. Therefore, we suggest that the cancer-protective effect of a sufficient vitamin D status may relate primarily to the ability to keep the immunocompetence of a person at a high level. Following the same argument, response index-adapted vitamin D sufficiency should (i) stabilize immune resilience, (ii) protect against numerous other diseases, and (iii) keep the rate of aging low. The latter is related to the low impact of the various hallmarks of aging, such as low levels of inflammation and other forms of cellular stress. Thus, vitamin D sufficiency is an important component of healthy aging, not only for keeping bone and skeletal muscle in good shape but also for the homeostasis of the immune system [70].

## 5. Conclusions

The biologically most active form of vitamin D, 1,25(OH)_2_D_3_, is an endocrine molecule that modulates in vitro and in vivo the epigenome of immune cells. We presented here a mechanism for how vitamin D affects the epigenetic programming of immune cells, in particular monocytes and their derived cell types. According to this concept, individuals with vitamin D sufficiency should have a higher level of immunocompetence than insufficiently supplemented people. A large number of observational studies have reported associations between vitamin D deficiency and increased risk for numerous diseases, as well as accelerated aging [71,72]. From our point of view, the unifying aspect of all these reports is the reduced immunocompetence of the investigated persons. Thus, elevating the immunocompetence of the concerned individuals, e.g., by shifting their vitamin D status to sufficiency, is of key importance.

When evaluating the vitamin D sufficiency of individuals with reduced immunocompetence, their vitamin D response index should also be taken into account since low vitamin D responders need more prominent vitamin D_3_ supplementation than high responders [17,19]. Because determining the vitamin D index of a person is more complex and expensive than measuring his/her vitamin D status, we suggest as a precaution that everyone should be supplemented as a low vitamin D responder, i.e., with a daily vitamin D_3_ dose of 1 µg (40 IU)/kg body mass. This dose is higher than the recommended dose for the average population in most countries [73], but still far below an amount that may cause side effects like hypercalcemia [74]. Please note that this supplementation guideline is based on our own experience and does not reflect any official recommendation.

## Data Availability

No new data were created or analyzed in this study. Data sharing is not applicable to this article.
